# *Streptomyces* Strains from Amazonian Sediments as Plant Growth Promoters and Biocontrol Agents of Anthracnose in Postharvest *Capsicum chinense*

**DOI:** 10.3390/microorganisms13122713

**Published:** 2025-11-28

**Authors:** Ingride Jarline Santos da Silva, Thiago Fernandes Sousa, Thayná Marães de Souza, Beatriz Miranda Gomes, Rudi Emerson de Lima Procópio, Aleksander Westphal Muniz, Rogério Eiji Hanada, Hector Henrique Ferreira Koolen, Gilvan Ferreira da Silva

**Affiliations:** 1 Programa de Pós-Graduação em Biotecnologia, Universidade Federal do Amazonas—UFAM, Manaus 69080-005, AM, Brazil; ingridejsantos@gmail.com (I.J.S.d.S.); fernandesthiago9620@gmail.com (T.F.S.); miranda.bia87@gmail.com (B.M.G.); 2 Embrapa Amazônia Ocidental—CPAA, Manaus 69010-970, AM, Brazil; thaynamaraes97@gmail.com (T.M.d.S.); alekswm@gmail.com (A.W.M.); 3 Programa de Pós-Graduação em Biotecnologia e Recursos Naturais, Universidade do Estado do Amazonas—UEA, Manaus 69065-001, AM, Brazil; rudiprocopio@gmail.com (R.E.d.L.P.); hkoolen@uea.edu.br (H.H.F.K.); 4 Instituto Nacional de Pesquisas da Amazônia—INPA, Manaus 69060-062, AM, Brazil; rhanadainpa@gmail.com

**Keywords:** biological control, *Streptomyces*, *Colletotrichum scovillei*, sustainable agriculture, amazon basin

## Abstract

Postharvest diseases represent a critical challenge for global agriculture, resulting in substantial economic losses and threatening worldwide food security. Species of the genus *Colletotrichum* stand out among the main phytopathogens for being responsible for up to 40% of postharvest losses in various crops, including *Capsicum* species. This study evaluated the antifungal activity of two *Streptomyces* strains isolated from Amazonian sediments against different *Colletotrichum* species, with a focus on *C. scovillei*, the causal agent of anthracnose in *Capsicum chinense* fruits. Multilocus phylogenetic analyses indicated that strain APUR 32.5 possibly represents a new species, while MPUR 40.3 was identified as *Streptomyces murinus*. Both strains exhibited in vitro antifungal activity against seven *Colletotrichum* species, with inhibition percentages ranging from 56.3% to 88.6%. In fruit bioassays, *S. murinus* MPUR 40.3 reduced the incidence of anthracnose by 95%, while *Streptomyces* sp. APUR 32.5 achieved a 39.25% reduction. Scanning electron microscopy revealed complementary mechanisms of antifungal action, with MPUR 40.3 acting during the early infection stages through germination tube lysis, while APUR 32.5 targeted established mycelial structures through hyphal degradation. Additionally, both strains demonstrated plant growth-promoting capacity and exhibited biotechnologically relevant characteristics, including production of hydrolytic enzymes, siderophores, and phosphate solubilization ability. These results highlight the biotechnological promise of these Amazonian isolates as multifunctional agents for the sustainable management of anthracnose in *Capsicum* peppers.

## 1. Introduction

Postharvest diseases threaten global food security, causing annual losses that exceed 1.3 billion tons and generating approximately US $200 billion in economic damage worldwide [1,2]. In developing countries, where storage infrastructure and disease management are often inadequate, reductions in yield can be as high as 78% in fruits, 54% in vegetables, and 32% in cereals, directly impacting farmers’ livelihoods and food availability [3,4]. In this context, phytopathogens emerge as a critical determinant that compromises global agricultural production, accounting for an estimated 30% to 40% reduction in the potential crop yield in various cultures.

The genus *Colletotrichum* encompasses phytopathogenic species that are capable of causing disease during both preharvest and postharvest stages, and affect numerous economically important plant species [5,6,7]. These pathogens exhibit genetic mechanisms that favor fungicide-resistant genotype selection under selective pressure, making long-term control particularly challenging [8,9,10]. The intensive use of fungicides creates directional selection pressure, which represents one of the main obstacles for sustainable disease management and highlights the need for integrated strategies that minimize resistance development while preserving the efficacy of control measures [11].

Anthracnose is among the most destructive diseases that affect pepper (*Capsicum* spp.) production in tropical and subtropical regions, and causes substantial losses during both field cultivation and postharvest storage [12]. *Colletotrichum scovillei* is responsible for the most destructive and prevalent manifestation of this disease in pepper crops. Although the first official report from Brazil of *C. scovillei* as the causal agent dates from 2014, recent evidence indicates systematic underestimation of its occurrence in pepper-producing regions [13,14]. This situation is further complicated by the growing evidence showing *C. scovillei* isolates with reduced fungicide sensitivity, resulting from intensive usage of chemical compounds [12,15]. This complex epidemiological scenario, characterized by widespread pathogen distribution and emerging resistant populations, demands alternative control strategies that are effective, sustainable, and capable of reducing resistance selection risk [16].

Among the biocontrol agents that are capable of sustainable phytopathogen management, *Streptomyces* bacteria distinguish themselves through their exceptional biosynthetic capacity and ecological versatility. These species produce vast arsenals of bioactive secondary metabolites and extracellular enzymes with antimicrobial activity, accounting for approximately 75–80% of all commercially used natural bioactive compounds, with over 100,000 documented antibiotics [17,18]. The identification of 279 new natural metabolites between 2015 and 2020 demonstrates the genus’s capacity as a source of biotechnological innovation for agricultural applications [18]. In agricultural contexts, *Streptomyces* performs multiple beneficial functions simultaneously: biological control of phytopathogens, promotion of plant growth, and soil health maintenance [19,20,21,22]. This multifunctionality is particularly relevant for developing microbial inoculants, as demonstrated by commercial biofungicides based on *S. griseoviridis* and *S. lydicus*. In addition to applications in biocontrol, these bacteria also contribute to nutrient cycling through complex organic residue degradation and participate in biofertilizer production, offering integrated approaches for sustainable agricultural systems [23,24].

Given the increasing use of agrochemicals in contemporary agriculture and the associated environmental impacts, the development of sustainable alternatives that can be effectively incorporated into agricultural practices becomes imperative [1,2,14].

The Amazon basin, a global hotspot of biodiversity, is characterized by its unique ecological pressures and unparalleled microbial diversity. It is suggested that this selective environment drives the evolution of microorganisms, such as *Streptomyces*, towards the production of novel and highly potent bioactive compounds, which may offer innovative solutions for agricultural challenges [25,26]. Therefore, we hypothesize that *Streptomyces* strains isolated from these underexplored Amazonian sediments may possess enhanced or distinct biocontrol mechanisms and plant growth-promoting capabilities. These traits could provide valuable alternatives for sustainable management of phytopathogens like *Colletotrichum scovillei*.

The present study investigated the antifungal activity and plant growth-promoting capabilities of two Amazonian *Streptomyces* strains (APUR 32.5 and MPUR 40.3) against *Colletotrichum* species, with a focus on *C. scovillei* as the main causal agent of pepper fruit anthracnose during the postharvest phases. To this end, our methodology included comprehensive in vitro antifungal assays, postharvest biocontrol evaluations on *Capsicum chinense* fruits, with mechanistic insights from scanning electron microscopy, and assessments of plant growth-promoting potential. We further characterized biotechnologically relevant traits like hydrolytic enzyme production, siderophore secretion, and phosphate solubilization, in addition to evaluating their ecophysiological versatility.

## 2. Materials and Methods

### 2.1. Sample Collection

Sample collection was carried out in December 2018 from sediments of the Purus River, located on the right bank of the Amazon River in the state of Amazonas, Brazil. Isolation of microorganisms from these samples was performed using the serial dilution technique at a concentration of 10^−3^. Inoculation was carried out on Petri dishes containing either AIA or ISP2 culture media (Table S1), supplemented with nalidixic acid (50 µg/mL) and cycloheximide (50 µg/mL). The plates were then incubated at 28 °C for 7 to 15 days. The streak plating technique was used to obtain pure cultures. Specifically, the isolate APUR 32.5 (*Streptomyces* sp., 06°39′54.0′′ S, 064°33′36.5′′ W, 5.5 m depth) was obtained from the AIA medium, and MPUR 40.3 (*Streptomyces* sp., 05°39′31.9′′ S, 063°38′53.7′′ W, 4.5 m depth) was obtained from the ISP2 medium. These isolates are currently part of the microbiological collection of the Laboratory of Amazon MicroBiotech–Embrapa Western Amazon, preserved in 15% (*v*/*v*) glycerol and stored at −80 °C [27].

### 2.2. Morphological Characterization

The phenotypic characteristics of the isolates were evaluated on different culture media (Table S1) at pH 7 under controlled temperature conditions (28 °C). The parameters analyzed included colony growth, aerial hyphae formation, vegetative mycelium development, and soluble pigment production. Observations were conducted over a 10-day period following the protocols established by the International *Streptomyces* Project [28]. Growth intensity was classified based on the morphological development: optimal growth (+++), characterized by abundant vegetative mycelium and aerial hyphae production throughout the entire inoculation line; moderate growth (++), with partial aerial mycelium formation and lower overall density; reduced growth (+), predominantly limited to vegetative mycelium with absent or scarce aerial hyphae; and no colony growth (−).

### 2.3. DNA Extraction

Isolates were cultured for 4 days in 50 mL Falcon tubes containing 20 mL of liquid ISP2 medium under agitation at 180 rpm and 28 °C. Total DNA extraction was performed using 2% CTAB cationic detergent [29]. DNA quantity was estimated by spectrophotometry (NanoDrop 2000, Thermo Scientific, Waltham, MA, USA), while integrity was assessed by electrophoresis on 0.8% (*w*/*v*) agarose gel.

### 2.4. PCR Amplification

PCR amplification was conducted using five primer pairs, targeting the partial sequences of *atpD* (ATP synthase F1, beta subunit), *gyrB* (DNA gyrase subunit B), *trpB* (tryptophan synthase beta subunit), *rpoB* (RNA polymerase beta subunit), and *recA* (recombinase A) genes. The primer sequences and annealing temperatures are described in Table S2, with PCR reactions performed in a final volume of 25 μL. This volume consisted of 2.5 μL EasyTaq^®^ 10X buffer (TransGen Biotech, Beijing, China), 10 μM dNTP, 1 U EasyTaq^®^ Taq DNA polymerase (TransGen Biotech, Beijing, China), 0.5 μM of each primer, 1 μL total DNA (50 ng), and autoclaved distilled water adjusted to achieve the final volume. Amplification conditions included: initial DNA denaturation at 95 °C for 3 min, followed by 35 cycles of denaturation at 95 °C for 15 s, primer-specific annealing for 30 s, and extension at 72 °C for 1.5 min. Final extension was performed at 72 °C for 5 min. The PCR products were resolved on 1.5% (*w*/*v*) agarose gel stained with ethidium bromide and documented using a molecular imaging system (Locus Biotechnology L-Pix. Chemi, São Paulo, Brazil). The amplified products were compared with a 1 kb plus size marker (Invitrogen, Carlsbad, CA, USA).

### 2.5. Sanger Sequencing

The PCR products were purified using ExoSAP-IT (Applied Biosystems, product code: 15819906, Foster City, CA, USA). For this procedure, 5 μL of PCR product and 2 μL of ExoSAP-IT were incubated at 37 °C for 15 min, followed by enzymatic inactivation at 80 °C for 15 min. The purified products were subjected to a BigDye terminator reaction in a total volume of 10 μL containing 2 μL of ultrapure water, 1.5 μL of 5X BigDye buffer, 0.5 μL of BigDye Terminator v3.1 (Thermo Fisher), 1 μL of each primer, and 5 μL of PCR products. The cycling conditions were: denaturation at 96 °C for 60 s, followed by 35 cycles at 96 °C for 15 s, 50 °C for 15 s, and 60 °C for 4 min. Reactions were resolved via capillary electrophoresis in a genetic analyzer (3500, Thermo Fisher).

### 2.6. Phylogenetic Analysis

Consensus sequences were obtained through the manual analysis of the generated electropherograms. Based on the dataset generated [30], *atpD* gene sequences from 335 different *Streptomyces* strains were retrieved, and neighbor-joining phylogenetic analysis was conducted to identify species closely related to strains APUR 32.5 and MPUR 40.3. Construction of the multilocus analysis dataset resulted in 40 *Streptomyces* strains plus the two strains identified in this study (Table S3). Sequences for each locus were individually aligned using the MAFFT algorithm and subsequently concatenated manually. The resulting file was processed on the IQ-TREE platform, and maximum likelihood (ML) analysis was conducted using 1000 bootstraps. The tree topology was visualized using the iTOL platform and manually edited with CorelDRAW version 2020 software.

### 2.7. In Vitro Antifungal Activity

The inhibition capacity of *Streptomyces* against seven *Colletotrichum* species was evaluated using the dual culture pairing technique [31], in which a 0.8 cm diameter mycelial disk of each phytopathogen was placed in the center of a plate containing PDA culture medium, and single streaks of selected isolates were made at the sides, 2 cm from the edges, followed by incubation at 28 °C. The phytopathogens used belong to the microbiological collection of Embrapa Western Amazon and are listed in Table S4. Tests were performed in triplicate, and evaluation was conducted through measurements of the colony diameter after 15 days, using the following calculation: % inhibition = [(C − T)/C] × 100, where C is the mean of the control and T corresponds to the mean of the treatment. For the positive control, only the phytopathogen in the culture medium was used.

### 2.8. Postharvest Biocontrol Activity

#### 2.8.1. Fruit Preparation and Pathogen Inoculum

*Capsicum chinense* fruits were surface-disinfected with 2% sodium hypochlorite for 5 min, followed by three rinses with sterile distilled water (2 min each) and rapid immersion in 70% ethanol. The inoculum of *C. scovillei* was prepared by harvesting conidia from 3-day-old cultures grown on potato dextrose medium. The conidia were suspended in sterile distilled water, and the concentration was adjusted to 10^8^ conidia/mL using a Neubauer counting chamber.

#### 2.8.2. Preparation and Standardization of Streptomyces Spore Suspension

The *Streptomyces* isolates APUR 32.5 and MPUR 40.3 were cultured on ISP2 agar medium for 7 days at 28 °C. Spores were collected by gently scraping the agar surface and then suspended in sterile distilled water. The spore suspension was standardized to a final concentration of 10^8^ CFU/mL by adjusting the optical density to 0.1 at 600 nm [32].

#### 2.8.3. Inoculation and Treatment Procedure

To evaluate the protective effect, fruits were first inoculated by immersion in the *C. scovillei* conidial suspension (10^8^ conidia/mL) for 5 s and incubated at 28 °C for 24 h to allow establishment of the pathogen. Subsequently, the fruits were treated by immersion in the *Streptomyces* spore suspension (10^8^ CFU/mL) for 5 s.

#### 2.8.4. Controls and Evaluation

Negative (sterile water) and positive (only *C. scovillei* inoculation) controls were included in the experiment. After 7 days of incubation at 28 °C, the disease index (DI) and control efficiency (CE) were calculated as follows: DI (%) = (lesion area/total fruit area) × 100, and control efficiency: CE (%) = [(IC − IT)/IC] × 100, where IC = positive control index and IT = treatment index. The experiment was performed in triplicate with 10 fruits per replicate.

### 2.9. Scanning Electron Microscopy

The structures of *Streptomyces* and their antagonistic interactions with the phytopathogen *C. scovillei* were analyzed via scanning electron microscopy (SEM) (JEOL, JSM-IT500HR, Akishima, Japan). For the morphological analysis, the microcultures were grown on ISP2 medium and incubated at 28 °C for 5 days. After 7 days, in vitro and postharvest interactions were evaluated under the same experimental conditions by removing fragments from the active margins of *C. scovillei* colonies. The samples were then fixed in 4% paraformaldehyde and dehydrated in a graded ethanol series (30%, 40%, 50%, 70%, 80%, 90% and 100%) for 15 min in each solution, repeating the absolute ethanol step twice. Finally, the microcultures were dried in a critical point dryer (Leica EM CPD300, Wetzlar, Germany) and sputter-coated with gold (DII-29010SCTR Smart Coater, Sono-Tek Corporation, Milton, NY, USA) for high-resolution image acquisition.

### 2.10. Evaluating Plant-Growth-Promotion Mechanisms

#### 2.10.1. Phosphate Solubilization and Siderophore Production

The ability of the isolates to solubilize different phosphate sources (Ca_3_(PO_4_)_2_, AlPO_4_, and FePO_4_) and to produce siderophores was evaluated using qualitative assays on selective and differential solid media (Table S5). Plates were incubated at 28 °C for 5 days, and solubilization or siderophore production was assessed based on the formation of halos around the colonies, indicating the diffusion of solubilized compounds or enzymes in the medium. All the tests were performed in triplicate to ensure reproducibility [33].

#### 2.10.2. Evaluation of Extracellular Enzyme Production

The production of amylase, cellulase, chitinase, lipase, and protease was evaluated via a qualitative assay using the enzymatic index (EI). The isolates were inoculated on plates containing differential media that were specific for each enzyme (Table S6) and then incubated for 7 days at three temperatures (25, 30, and 35 °C) and three pH values (4, 7, and 10). The enzymatic index was calculated as the ratio between the mean diameter of the substrate hydrolysis halo (dh) and the mean colony diameter (dc), both measured in millimeters, according to the equation EI = dh/dc. The bioassays were performed in triplicate, and for each isolate, the enzymatic activity was expressed as the ratio between the degradation halo and colony growth [34].

#### 2.10.3. pH and Temperature Tolerance

Isolate tolerance was evaluated on ISP2 medium, with pH adjusted to 6, 7, and 8, and incubated at temperatures of 25, 30, 35, 40, 45, and 50 °C. The growth of the isolates was monitored over a seven-day incubation period, and the optimal cultivation conditions were determined based on the growth performance, following the methodology described in Section 2.2.

### 2.11. Growth Promotion of Capsicum Chinense

The plant growth-promoting capacity of the *Streptomyces* isolates was evaluated using *Capsicum chinense* plants under controlled conditions.

#### 2.11.1. Plant Preparation and Cultivation

The *Capsicum chinense* seeds were surface-sterilized by immersion in 70% ethanol for 2 min, followed by 2% sodium hypochlorite for 5 min, and subsequently rinsed four times with sterile distilled water. After sterilization, the seeds were sown in seedling trays and cultivated for 30 days. Subsequently, the seedlings were transplanted into individual 1000 mL pots containing Vivato Plus substrate. Each pot contained a single plant. The seedlings were irrigated twice daily (morning and afternoon). Every 20 days after transplantation, 50 mL of SARRUGE nutrient solution was added to each pot, consisting of: Ca(NO_3_)_2_·4H_2_O—1180 mg/L; KNO_3_—505 mg/L; MgSO_4_·7H_2_O—493 mg/L; KH_2_PO_4_—272 mg/L; (NH_4_)_2_SO_4_—230 mg/L [34].

#### 2.11.2. Bacterial Inoculum Preparation and Application

For the preparation of the inoculum, the *Streptomyces* strains were cultured in ISP2 broth at 28 °C under constant agitation (120 rpm) for 5 days. The bacterial suspension was standardized to a final concentration of 10^8^ CFU/mL by adjusting the optical density to 0.1 at 600 nm [32]. Seven days after transplantation (30 days after germination), 20 mL of the bacterial cell suspension was applied to the soil near the stem base of each plant.

#### 2.11.3. Experimental Design, Controls, and Growth Evaluation

The experiment included treatments with each *Streptomyces* isolate (APUR 32.5 and MPUR 40.3) and a non-inoculated control group, with 10 pots per treatment. The control plants received the same volume of sterile ISP2 broth (without bacteria). On the 75th day after transplantation, the plants were harvested for growth evaluation. Parameters assessed included shoot length, root length, and dry mass. Dry mass was determined after drying all plant material in an oven at 50 °C until constant weight was achieved [34].

### 2.12. Statistical Analysis

Data obtained from antagonism assays (in planta and in vitro), enzymatic analyses, and plant growth promotion tests were subjected to analysis of variance (ANOVA) using R v4.1.3 software (R Core Team, Vienna, Austria, 2022). For comparison of the means, the Scott-Knott test was applied to the results of the in vitro antagonism assays and the Dunn test for postharvest analysis (in planta). The significance level used was *p* ≤ 0.05.

## 3. Results

### 3.1. Morphological Aspects and Molecular Identification

#### 3.1.1. Morphological Characterization

**Figure 1:**
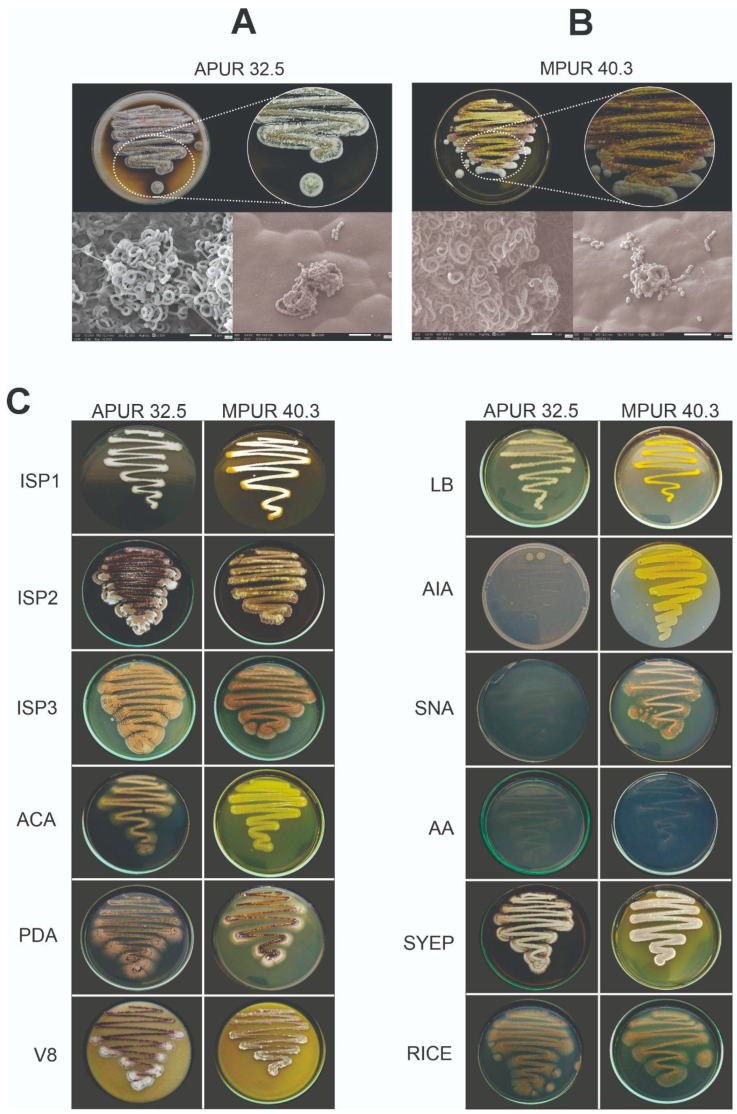
Morphological and micromorphological characteristics of *Streptomyces* isolates on different culture media. (**A**) Macroscopic colonial aspect of *Streptomyces* sp. APUR 32.5 grown on ISP2 medium (pH 7, 40 °C, 7 days) and its corresponding micromorphology (spiraled spore chains with cubic, wrinkled spores). (**B**) Macroscopic colonial aspect of *Streptomyces murinus* MPUR 40.3 grown on ISP2 medium (pH 7, 30 °C, 7 days) and its corresponding micromorphology (well-developed vegetative and aerial mycelia forming closed spiral spore chains with cubic, smooth spores). (**C**) Comparative overview of the morphological characteristics of *Streptomyces* sp. APUR 32.5 and *Streptomyces murinus* MPUR 40.3 isolates cultivated on twelve different culture media after 7 days of incubation at 28 °C, highlighting variations in mycelial coloration and diffusible pigment production.

The *Streptomyces* sp. APUR 32.5 and *S. murinus* MPUR 40.3 isolates were characterized on twelve distinct culture media and exhibited morphological features typical of the *Streptomyces* genus. Phenotypic variations were observed depending on the specific medium used (Figure 1A,B).

For the *Streptomyces* sp. APUR 32.5 isolate, optimal growth (+++) was recorded on ISP2, ISP3, SYEP, V8, and rice agar media. Moderate growth (++) occurred on ISP1, ACA, and PDA, while limited growth (+) was noted on LB, SNA, AA, and AIA. Colony mycelial coloration predominantly ranged from white to brown. Notably, a black diffusible pigment was produced specifically on SYEP medium. Microscopic analysis of APUR 32.5 revealed spiraled spore chains, with the spores exhibiting a cubic shape, a wrinkled surface, and an approximate size of 0.13 μm (Figure 1A,B).

The *S. murinus* MPUR 40.3 isolate showed optimal growth (+++) on ISP2, ISP3, V8, SYEP, SNA, and rice agar. Moderate growth (++) was observed on ISP1 and PDA, and limited growth (+) was observed on ACA, LB, AIA, and AA. A distinct characteristic of MPUR 40.3 was the prominent production of a yellow diffusible pigment in eight of the twelve tested media (ISP1, ISP2, ACA, PDA, LB, AIA, V8, and SYEP). The aerial mycelium displayed varied colorations, including brown, yellow, lilac, and white tones, depending on the culture medium. Microscopic evaluation of MPUR 40.3 demonstrated well-developed vegetative and aerial mycelia forming closed spiral spore chains, with spores that were cubic in shape and had a smooth surface, and measured approximately 0.15 μm (Figure 1A,B).

#### 3.1.2. Molecular Identification and Phylogenetic Analysis

Molecular identification of the isolates was performed through multilocus phylogenetic analysis based on the concatenated alignment of five housekeeping genes (*atpD*, *gyrB*, *recA*, *rpoB*, and *trpB*) from 40 *Streptomyces* species that are closely related to APUR 32.5 and MPUR 40.3. The final alignment comprised 2529 characters, including gaps, distributed among the genes *atpD* (496 bp), *gyrB* (417 bp), *recA* (504 bp), *rpoB* (541 bp), and *trpB* (541 bp).

**Figure 2:**
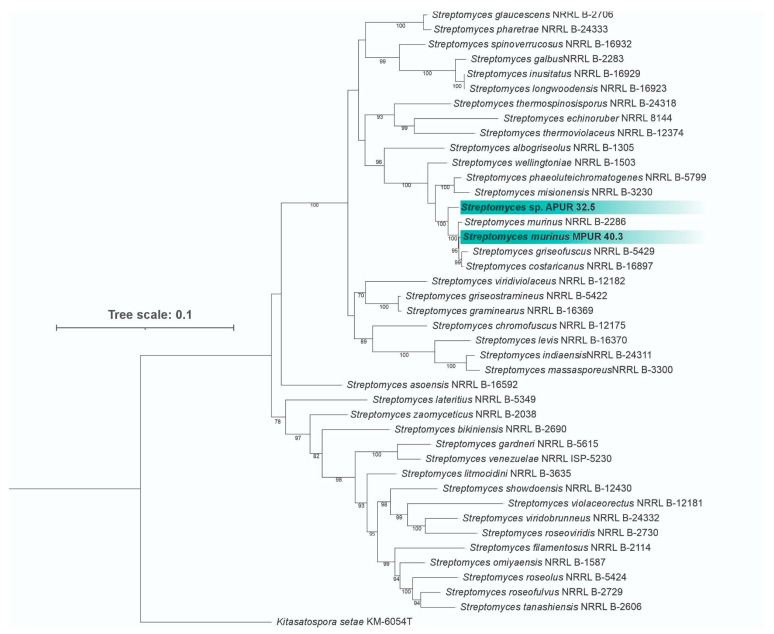
Phylogenetic tree based on multilocus analysis (*atpD*, *gyrB*, *recA*, *rpoB*, and *trpB*) of *Streptomyces* sp. APUR 32.5 and *S. murinus* MPUR 40.3 (highlighted in green) and related species. The tree was constructed using the maximum likelihood method with 1000 bootstrap replicates, using *Kitasatospora setae* KM-6054T as an outgroup. Numbers on the nodes indicate bootstrap support values (%).

The most suitable evolutionary models to explain the data according to the AIC criteria were GTR + F + I + G4 for the *atpD*, *recA*, and *trpB* genes, GTR + F + G4 for the *gyrB* gene, and K3Pu + F+I + G4 for *rpoB*. The phylogenetic tree obtained by maximum likelihood estimation with 1000 bootstrap replicates revealed that the isolate MPUR 40.3 clustered with high support (99% bootstrap) in the clade including *S. murinus* DSM 40091T, confirming its identification as belonging to this species (Figure 2).

Isolate APUR 32.5 formed a distinct branch, yet was closely related to the clade that includes *S. murinus*, *S. griseofuscus*, and *S. costaricanus* (currently considered heterotypic synonyms of *S. murinus*), suggesting that this isolate represents a putative new species for the genus.

### 3.2. Antifungal Activity

**Figure 3:**
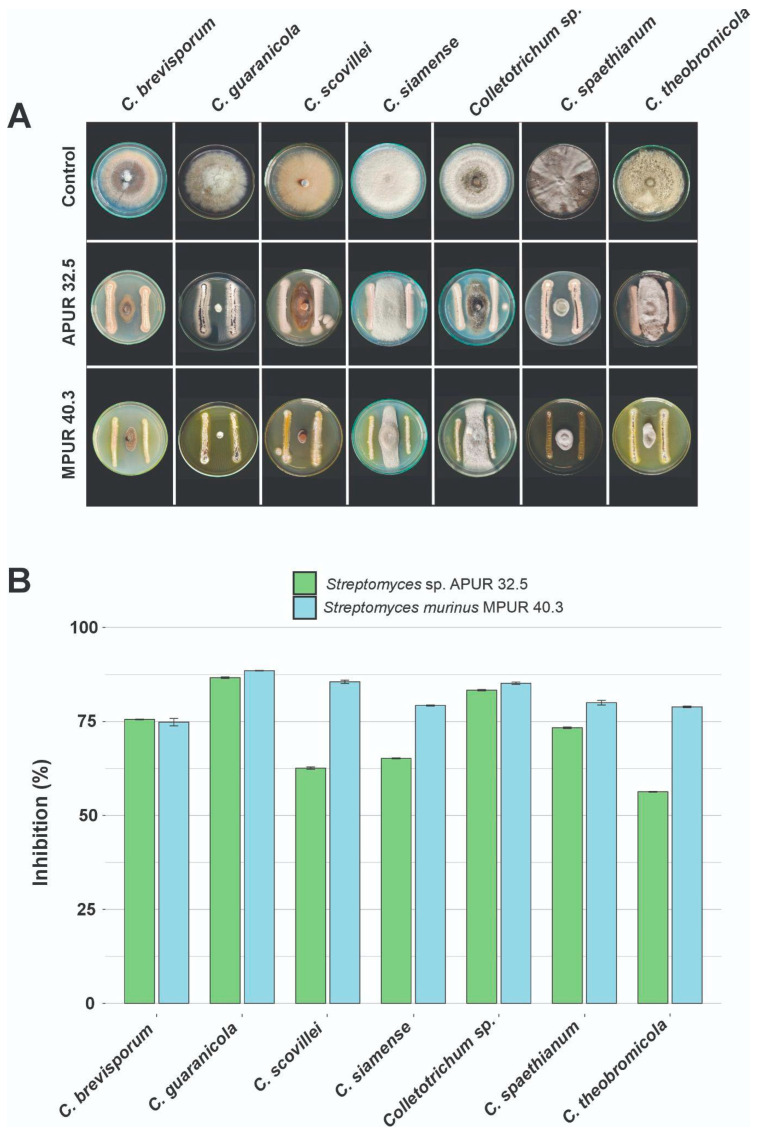
In vitro antifungal activity of *Streptomyces* isolates against *Colletotrichum* species. (**A**) Representative images of direct antagonism assays on Petri dishes, demonstrating mycelial growth inhibition of seven *Colletotrichum* species by *Streptomyces* sp. APUR 32.5 and *S. murinus* MPUR 40.3 isolates. (**B**) Bar chart representing the percentage of mycelial growth inhibition (PIC) of the tested phytopathogens after 7 days of cultivation at 28 °C. Bars indicate mean values, and error bars represent the standard deviation of three replicates.

The results indicated that both *Streptomyces* strains demonstrated antifungal activity against the seven *Colletotrichum* species analyzed, with inhibition percentages ranging from 56.3% to 88.6% (Figure 3A,B). *Streptomyces* sp. APUR 32.5 showed the highest inhibition rates against *C. guaranicola* (INPA 2939), reaching 86.7 ± 0.2%, *Colletotrichum* sp. (INPA 2973) with 83.3 ± 0.17%, and *C. brevisporum* (INPA 2787) with 75.6 ± 0.1%. In turn, *S. murinus* MPUR 40.3 demonstrated greater efficiency in inhibiting the mycelial growth of *C. guaranicola* (INPA 2939) with 88.5 ± 0.05%, *C. scovillei* (INPA 2910) with 85.6 ± 0.43%, and *Colletotrichum* sp. (INPA 2973) with 85.2 ± 0.3% (Figure 3B). All the tested isolates exhibited inhibition percentages over 50%, confirming the broad spectrum of these strains against the evaluated species.

**Figure 4:**
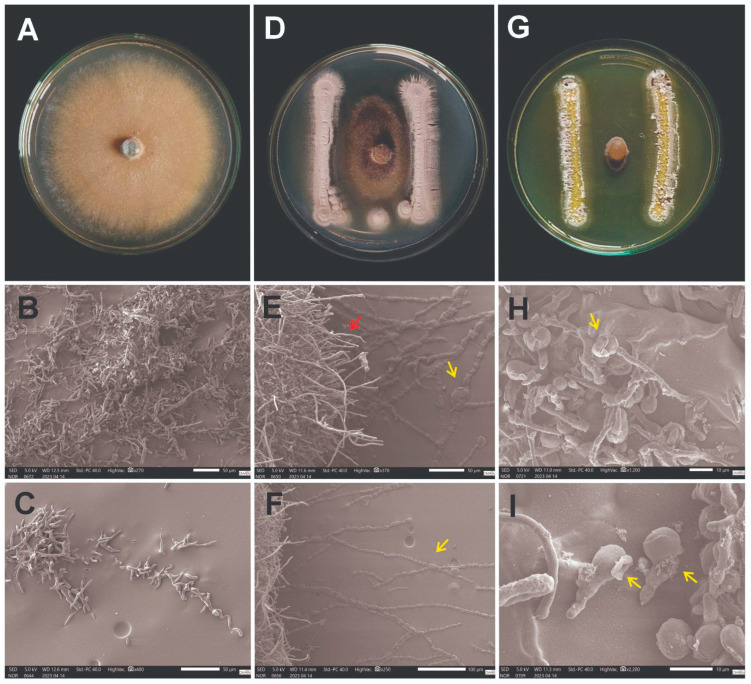
Images of the interaction between *Streptomyces* spp. and *Colletotrichum scovillei* using scanning electron microscopy (SEM). (**A**–**C**) Control: pure culture of *Colletotrichum scovillei* showing normal growth and mycelia with preserved structures, continuous hyphae of uniform diameter and smooth surface. (**D**–**F**) Interaction between *Streptomyces* sp. APUR 32.5 and *Colletotrichum scovillei*: morphological alterations in phytopathogen mycelia are evident, with irregular hyphal thinning and cell wall disruption (yellow arrow), while some hyphae preserve a morphology that is similar to the control (red arrow). (**G**–**I**) Interaction between *Streptomyces murinus* MPUR 40.3 and *Colletotrichum scovillei*: cellular lysis of *C. scovillei* conidia is observed during the germination phase, resulting in structural collapse (yellow arrows) and inhibition of germination. Scale bars are shown in the SEM images.

Scanning electron microscopy analyses revealed the differential morphological effects of the *Streptomyces* strains on the *C. scovillei* structures. Control cultures containing only the pathogen (Figure 4A–C) displayed intact hyphal networks with a uniform diameter and a smooth surface morphology. In dual cultures with *Streptomyces* sp. APUR 32.5 (Figure 4D–F), *C. scovillei* hyphae exhibited morphological alterations that appeared to correlate with proximity to the interaction zone. Hyphae closer to the bacterial colonies showed cell wall disruption and irregular thinning (yellow arrow, Figure 4E), whereas hyphae in more distant regions maintained relatively normal morphology (red arrow, Figure 4E), suggesting a concentration-dependent effect of the diffusible compounds.

Co-cultures with *S. murinus* MPUR 40.3 (Figure 4G–I) exhibited distinct morphological patterns, with *C. scovillei* conidia showing structural collapse and evident signs of lysis (Figure 4H,I). Although the damage patterns differed from those observed for APUR 32.5, these observations indicate that both isolates inhibit the pathogen through antibiosis. This inference is supported by the absence of direct contact between the antagonist and the fungal structures, together with the presence of diffusible factors causing visible cellular disruption. While the specific metabolites responsible cannot be identified based on SEM images, the results clearly demonstrate that MPUR 40.3 exerts a more pronounced inhibitory effect, preventing the fungal hyphae from approaching the bacterial colony.

### 3.3. Effect of Streptomyces Isolates on the Control of Colletotrichum Scovillei in Capsicum Chinense Fruits

#### 3.3.1. Evaluation of the Incidence of Disease

**Figure 5:**
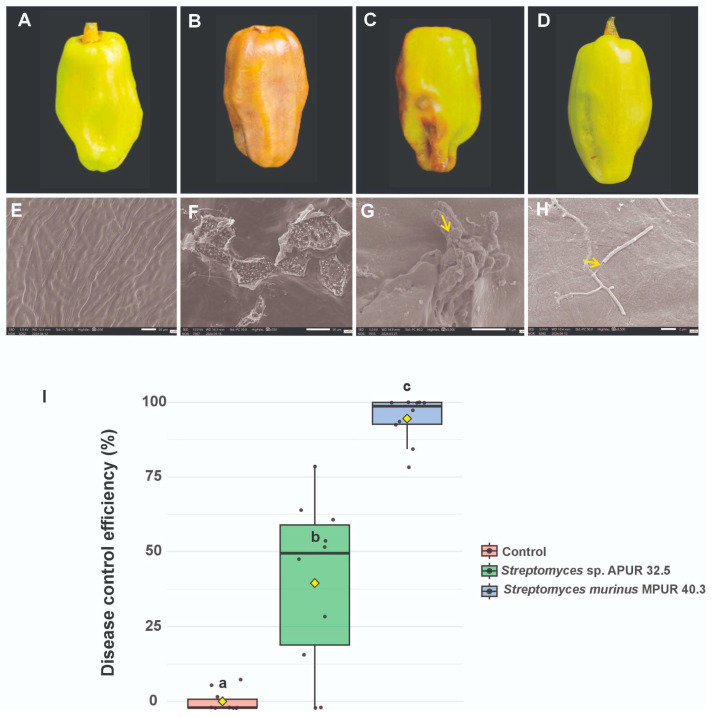
Evaluation of the interaction between *Streptomyces* and *Colletotrichum scovillei* on pepper fruits and ultrastructural analysis of infected tissue surfaces. Macroscopic images of fruits: (**A**–**E**) Negative control, showing the intact epicarp of the fruit. (**B**–**F**) Positive control, evidencing fungal colonization and fruit surface rupture. (**C**–**G**) Fruit treated with *Streptomyces* sp. APUR 32.5, with reduced lesions and adherence of antagonist spores to pathogen conidia (yellow arrow indicating *Streptomyces* spores). (**D**–**H**) Fruit treated with *S. murinus* MPUR 40.3, with tissue preservation and structural collapse of *C. scovillei* hyphae (yellow arrow indicating broken mycelia). (**I**) Box plot showing the disease control efficiency (%) in different treatments. Means followed by different letters indicate statistically significant differences (*p* < 0.05).

After seven days of incubation (at 28 °C), the negative control fruits (treated with sterile water) remained completely healthy, with an intact surface and the absence of any signs of microbial colonization, as confirmed by SEM (Figure 5A–E). In contrast, the positive control fruits (inoculated only with *C. scovillei* INPA 2010) developed severe symptoms of anthracnose (Figure 5B), characterized by depressed necrotic lesions and abundant sporulation, reaching a disease index of 97.79% (Figure 5F).

The application of biocontrol agents resulted in significant protection, albeit with differential efficacy between strains. Fruits treated with *S. murinus* MPUR 40.3 exhibited high protection, with an infection index of only 4.86%, representing 95% control efficacy relative to the positive control group (Figure 5D–H). Fruits treated with *Streptomyces* sp. APUR 32.5 showed intermediate protection, with a disease incidence of 49.41% (Figure 5C–G).

Statistical analysis confirmed significant differences (*p* < 0.05) between all treatments, demonstrating the superior performance of *S. murinus* MPUR 40.3 as a biocontrol agent (Figure 5I). Importantly, additional controls in which fruits were treated exclusively with *Streptomyces* strains (without the pathogen) showed no morphological alterations or tissue damage.

#### 3.3.2. Ultrastructural Analysis of the Pathogen-Antagonist Interactions

Scanning electron microscopy enabled the elucidation of the antagonist mechanisms of action and their interaction with the pathogen in host tissues. In the positive control fruits, SEM revealed severe tissue surface damage with extensive fungal colonization and disruption of cellular integrity (Figure 5D). At higher magnifications, pathogen hyphae were observed actively penetrating fruit tissues (Figure 5C, yellow arrow), characterizing the typical infectious process of anthracnose.

In fruits treated with *Streptomyces* sp. APUR 32.5, the lesions on the plant tissue surfaces appeared less intense and less extensive compared with the positive control, although fruit damage was still observable. The micrographs evidenced a direct interaction between the antagonist and the pathogen, with *Streptomyces* sp. APUR 32.5 spores adhered to the conidia (Figure 5E, yellow arrow indicating conidium with *Streptomyces* spores on its surface), confirming the physical contact between the microorganisms. However, some conidia showed morphological alterations with apparent cell wall integrity compromise (Figure 5F, indicated by the yellow arrow), suggesting that the *Streptomyces* strain may also possess compounds capable of damaging the phytopathogen cell wall.

Treatment with *S. murinus* MPUR 40.3 resulted in significantly superior protection, with the fruit tissue surface predominantly preserved. Although the macroscopic analysis revealed minimal symptoms, SEM detected small, localized lesions, probably resulting from the initial 24-h pathogen exposure period before application of the antagonist. In the interaction areas, morphological alterations in *C. scovillei* hyphae were observed, characterized by structural disruption and mycelial disintegration (Figure 5H, indicated by a yellow arrow), demonstrating the potent direct antifungal activity of *S. murinus* MPUR 40.3.

These ultrastructural results corroborate the data obtained in the in vitro assays (Section 3.2), in which *S. murinus* MPUR 40.3 also demonstrated greater antagonistic activity against *C. scovillei*, and suggest that the biocontrol mechanisms involve both direct interaction and the possible production of antifungal compounds that compromise the pathogen’s structural integrity.

### 3.4. Plant Growth Promotion

The evaluation of the capacity for plant growth promotion by *Streptomyces* isolates was conducted through the analysis of multiple biometric parameters in *Capsicum chinense* plants cultivated under controlled conditions for 45 days after bacterial inoculation.

**Figure 6:**
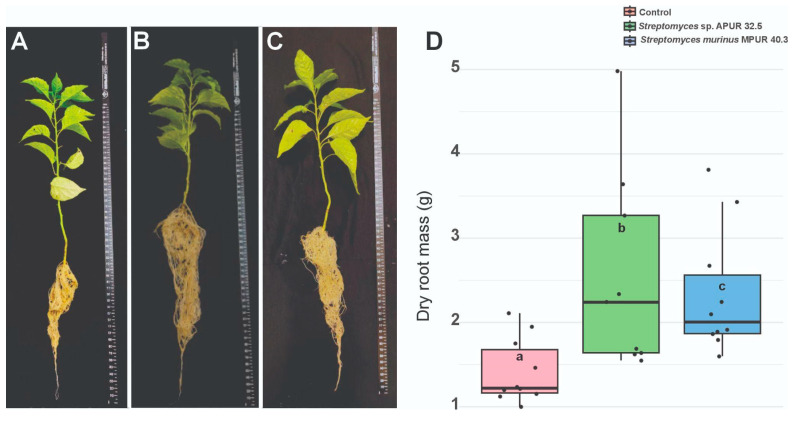
Effect of *Streptomyces* isolates on *Capsicum chinense* root system development after 45 days of cultivation. (**A**) Root system of control plants (uninoculated); (**B**) Root system of plants inoculated with *Streptomyces* sp. APUR 32.5; (**C**) Root system of plants inoculated with *Streptomyces murinus* MPUR 40.3; (**D**) Mean root dry weight (g) per treatment. Bars represent mean ± standard deviation (n = 10). Different letters above the bars indicate statistically significant differences between treatments by Tukey’s test (*p* < 0.05).

The results demonstrated that inoculation with *Streptomyces* sp. APUR 32.5 and *S. murinus* MPUR 40.3 promoted significant alterations in plant root system development, whereas shoot parameters showed no statistically significant differences (Figure 6). Root dry weight analysis revealed substantial increases in treatments with both bacterial isolates compared with the uninoculated control.

Plants treated with *Streptomyces* sp. APUR 32.5 showed the greatest increment in root dry weight, with a 79.58% increase relative to the control (*p* < 0.05), reaching an average of 0.87 ± 0.12 g per plant, while the control averaged 0.48 ± 0.09 g (Figure 6D). Treatment with *S. murinus* MPUR 40.3 also resulted in a significant increase, with a 64.08% increment in root dry weight (0.79 ± 0.11 g) compared to the control.

Morphological analysis of the root system (Figure 6A–C) evidenced not only an increase in biomass but also alterations in root architecture, with greater branching and lateral root development in the plants treated with *Streptomyces*, particularly with strain APUR 32.5. These modifications in the root architecture may contribute to improved water and nutrient uptake efficiency, representing an additional benefit beyond the increase in biomass.

Although no statistically significant differences were detected in plant height, stem diameter, or shoot dry weight, the substantial root development observed in the plants treated with *Streptomyces* suggests the suitability for application of these isolates as growth-promoting inoculants in *C. chinense*, especially under conditions where root development represents a limiting factor for productivity.

### 3.5. Enzymatic Assays

#### 3.5.1. Hydrolytic Enzymatic Activities

**Figure 7:**
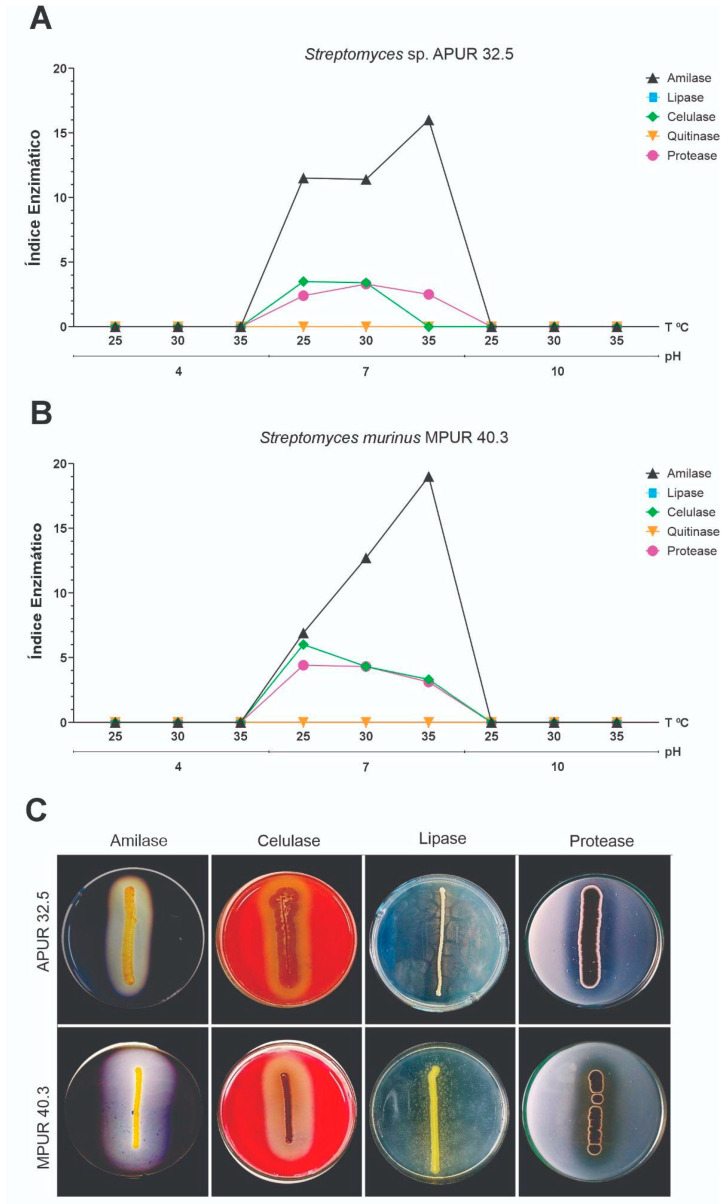
Hydrolytic enzymatic activities of *Streptomyces* isolates under different temperature and pH conditions. (**A**) Enzymatic indices (EI) of *Streptomyces* sp. APUR 32.5 for amylase, cellulase, and lipase under different temperature combinations. (**B**) Enzymatic indices of *Streptomyces murinus* MPUR 40.3 under the same conditions. (**C**) Representative visualization of enzymatic degradation halos.

Both isolates demonstrated the capacity to produce diverse hydrolytic enzymes, with significant variations in enzymatic indices (EI) as a function of temperature and pH conditions (Figure 7A-C). *Streptomyces* sp. APUR 32.5 exhibited notable amylolytic (EI = 16.0 ± 3.46) and lipolytic (EI = 10.0 ± 2.36) activities, particularly at pH 7.0 and 28 °C (Figure 7A). In contrast, *S. murinus* MPUR 40.3 displayed superior amylolytic activity (EI = 19.0 ± 0.00) under the same conditions, in addition to moderate cellulolytic (EI = 5.9 ± 1.18) and lipolytic (EI = 5.8 ± 0.70) activities (Figure 7B).

Comparative analysis of enzymatic activities under different conditions revealed that both isolates maintained significant hydrolytic activity across a broad pH range (5.0–9.0) and temperature range (25–37 °C), with optimal activity under neutral to slightly alkaline conditions (pH 7.0–8.0) and mesophilic temperatures (28–30 °C). This versatility suggests adaptability to different rhizosphere microenvironments and application under diverse edaphoclimatic conditions.

#### 3.5.2. Siderophore Production and Phosphate Solubilization

The capacity to mobilize essential nutrients was evaluated through qualitative assays for the siderophore production and solubilization of different phosphate sources. Both isolates, *Streptomyces* sp. APUR 32.5 and *S. murinus* MPUR 40.3 demonstrated positive results for siderophore production, as evidenced by the characteristic halo formation on CAS (chrome azurol S) medium.

Regarding phosphate solubilization, both isolates showed the capacity to mobilize phosphorus from diverse inorganic sources, including aluminum phosphate (AlPO_4_), tricalcium phosphate [Ca_3_(PO_4_)_2_], and ferric phosphate (FePO_4_). This versatility in solubilizing different forms of insoluble phosphate is particularly relevant, considering that phosphorus frequently represents a limiting factor for plant growth in tropical soils.

The combined capacity to produce siderophores and solubilize phosphates constitutes an important mechanism for plant growth promotion, contributing to the increased availability of essential nutrients in the rhizosphere. In addition, siderophore production may act as an indirect biocontrol mechanism through which phytopathogens compete for iron.

### 3.6. pH and Temperature Tolerance

The capacity to adapt to different environmental conditions is a crucial parameter for evaluating the application of microorganisms as biocontrol agents and plant growth promoters in the field. In this context, the characterization of the tolerance of *Streptomyces* isolates to different temperatures and pH values was performed, aiming to determine their ecophysiological versatility. *Streptomyces* sp. APUR 32.5 and *S. murinus* MPUR 40.3 isolates were evaluated for growth on ISP2 medium under different temperature (25, 30, 35, 40, 45, and 50 °C) and pH (6.0, 7.0, and 8.0) combinations.

*Streptomyces* sp. APUR 32.5 proved to be a moderately thermotolerant mesophile, with optimal growth (+++) at 35 °C across all tested pH ranges. At lower temperatures (25–30 °C), it showed moderate growth (++) regardless of pH. At 40 °C, it exhibited optimal growth (+++) at pH 6.0, reducing to moderate (++) under neutral to alkaline conditions (pH 7.0–8.0). At an elevated temperature (45 °C), growth was reduced (+) at all pH values, and at 50 °C, no growth was observed, indicating its upper thermal tolerance limit.

*S. murinus* MPUR 40.3 presented a slightly distinct growth profile, with a preference for more alkaline conditions at lower temperatures. At 25 °C, it demonstrated optimal growth (+++) at pH 8.0 and moderate growth (++) at pH 6.0–7.0. At temperatures of 30–35 °C, it exhibited optimal growth (+++) at pH 7.0–8.0 and moderate growth (++) at pH 6.0. At 40 °C, growth was moderate (++) under all pH conditions. Similarly to the isolate APUR 32.5, it showed reduced growth (+) at 45 °C and absence of growth at 50 °C.

Both isolates demonstrated the capacity to grow across a broad temperature range (25–45 °C) and pH range (6.0–8.0), with distinct preferences reflecting specific ecophysiological adaptations. *Streptomyces* sp. APUR 32.5 showed greater tolerance to elevated temperatures under slightly acidic conditions (pH 6.0), while *S. murinus* MPUR 40.3 demonstrated better adaptation to alkaline conditions (pH 8.0) at lower temperatures.

This ecophysiological versatility suggests the application of these isolates under different edaphoclimatic conditions, particularly in tropical and subtropical regions where soil temperatures can vary significantly. Additionally, tolerance to different pH values broadens their application in soils with diverse chemical characteristics.

## 4. Discussion

The *Streptomyces* strains investigated in this study show phylogenetic proximity to the clade that includes *S. murinus*, which is the currently accepted name for this species, while *S. griseofuscus* and *S. costaricanus* are considered heterotypic synonyms of *S. murinus* according to recent taxonomic revisions [35]. Strain MPUR 40.3 was identified as *S. murinus*, whereas *Streptomyces* sp. APUR 32.5 represents a distinct sister group, a new putative species within the genus. Complementary phylogenomic analyses (Table S7), including digital DNA-DNA hybridization (dDDH) values below the 70% cutoff for species delineation, further support the phylogenetic results and confirm the distinct taxonomic position of APUR 32.5.

*S. murinus* was originally described by Frommer in 1959 and is known for its ability to produce the antifungal metabolite pentamycin [36,37], first isolated in 1958 by Umezawa and collaborators. This compound exhibits activity against various pathogenic fungi, including *Candida* species and dermatophytes. The strain previously classified as *S. costaricanus*, described in 1995, is recognized for its antibiotic and anti-nematode properties, and is known to produce avermectin [38], a compound also produced by *Streptomyces avermitilis* [39]. The taxonomic reclassification of these species as *S. murinus* does not diminish the importance of their bioactive metabolites but rather underscores the complexity of *Streptomyces* taxonomy and the need for polyphasic approaches for accurate identification.

In the present study, the strains *Streptomyces* sp. APUR 32.5 and *S. murinus* MPUR 40.3 demonstrated strong in vitro antagonistic activity against seven distinct *Colletotrichum* species. Among the species tested, the best performance was observed against *C. guaranicola*, with inhibition of mycelial growth of over 80%, surpassing previously reported results, in which *Streptomyces griseocarneus* R132 reduced the mycelial growth of *C. guaranicola* by 70% [34]. Similarly, inhibition rates of up to 66% were reported against *C. siamense* using *Streptomyces* species [40]; whereas, in our study, the APUR 32.5 and MPUR 40.3 strains inhibited the growth of *C. siamense* by 65% and 79%, respectively. This inhibitory effect may be associated with the production of secondary metabolites, as *Streptomyces* is well-known for its remarkable ability to synthesize a wide array of bioactive compounds [41,42,43].

In addition to the direct action of microorganisms, *Streptomyces* extracts have also shown high efficacy against phytopathogens. *S. murinus* NARZ reduced the mycelial growth of *C. scovillei* by up to 65%, indicating the presence of secondary metabolites with significant antifungal activity [44]. Similarly, both the extract and the direct action of *Streptomyces* sp. NEAU-Y11 resulted in significant inhibition of the mycelial growth of *Colletotrichum orbiculare* [45], reinforcing the potential of this genus as a source of bioactive molecules with antifungal properties. Moreover, metabolites produced by *Streptomyces amritsarensis* V31 exhibited relevant antifungal activity in the control of different phytopathogenic fungi [46].

In addition to bioactive metabolites, hydrolytic enzymes also represent a promising alternative for controlling phytopathogens. Enzymes such as amylase, cellulase, lipase, glucanase, chitinase, and protease play an essential role in degrading the cell wall, proteins, and structural components of phytopathogenic fungi, establishing an efficient biocontrol mechanism that indirectly supports plant development [47,48]. In the present study, the tested *Streptomyces* strains demonstrated amylolytic, cellulolytic, lipolytic, and proteolytic activity, suggesting the involvement of these enzymes in the degradation of fungal structures and the suppression of phytopathogens.

The enzymatic index (EI) values for amylase reached significant levels, with *S. murinus* MPUR 40.3 (EI = 19) and *Streptomyces* sp. APUR 32.5 (EI = 16) standing out. These results indicate a high capacity for hydrolytic enzyme production, which is possibly associated with the biocontrol mechanism of these strains. The role of enzymes in microbial antagonism is supported by studies that demonstrate the importance of α-amylase (AmyS) produced by *Bacillus cereus* 0–9 in inhibiting *Rhizoctonia cerealis*. Through gene editing, the authors observed that the growth of the phytopathogen was reduced by 84.7% in the presence of the strain carrying the functional gene; however, deletion of the same gene (*amyS*) resulted in a significant decrease in biocontrol efficacy, with inhibition reduced to only 43.8% [49].

The postharvest control of anthracnose using microorganisms and their metabolites is increasingly recognized as an effective biocontrol strategy, crucial for reducing chemical inputs and promoting sustainable agriculture [43]. Our study significantly contributes to this understanding by demonstrating the strong postharvest biocontrol activity of *Streptomyces* sp. APUR 32.5 and *S. murinus* MPUR 40.3 against *C. scovillei* in *C. chinense* fruits (Section 3.3.1). Specifically, *S. murinus* MPUR 40.3 achieved a 95% reduction in anthracnose incidence, while *Streptomyces* sp. APUR 32.5 showed a moderate 39.25% reduction. These findings are highly encouraging and align with the growing body of evidence supporting the efficacy of microbial agents for postharvest disease management. For instance, the significant efficacy of MPUR 40.3 (95%) is comparable to, or even surpasses, some previous reports; as volatile organic compounds from *Trichoderma agriamazonicum* achieved complete inhibition against *C. scovillei* in *C. chinense* [50]. *Streptomyces tuirus* AR26 [51], *Streptomyces lactacystinicus* ZZ-84 [52], and *Streptomyces olivoreticuli* ZZ-21 [53] demonstrated growth inhibition of *Colletotrichum scovillei* both in vitro and in the assay of disease suppression in planta, and are reported as promising biocontrol strains that are capable of significantly reducing postharvest anthracnose caused by *C. scovillei*. This reinforces that diverse microbial groups, including *Streptomyces*, are robust alternatives to chemical inputs. Our results thus underscore the promising role of *Streptomyces* from Amazonian sediments as potent biocontrol agents for anthracnose, contributing to more sustainable agricultural practices.

SEM analyses revealed marked differences in the mechanisms of action of the antagonistic strains against *Colletotrichum scovillei*, both in vitro and postharvest biocontrol. In vitro treatment with *Streptomyces* sp. APUR 32.5 resulted in intense degradation of the pathogen’s mycelia, suggesting the production of lytic enzymes that compromise fungal cell wall integrity. In contrast, *S. murinus* MPUR 40.3 appeared to release substances that acted directly on germinative cells (conidia), promoting their destruction and consequently preventing germination and establishment of infection.

Postharvest assays showed that, despite the moderate reduction in disease severity provided by strain APUR 32.5, SEM revealed visibly damaged and shriveled *C. scovillei* spores on the surface of the treated fruits. In contrast, in the treatment with MPUR 40.3, the spores were completely ruptured, corroborating the greater efficacy of this strain. The use of SEM as a tool to visualize interactions between antagonistic microorganisms and pathogens has proven fundamental for elucidating biocontrol mechanisms, as shown in previous studies that revealed structural alterations in different pathogen–antagonist systems [54,55].

The *Streptomyces* strains tested in this study demonstrated multifunctional activity, acting not only in the biocontrol of phytopathogens but also in the promotion of plant growth. Both isolates were able to produce siderophores and solubilize different forms of phosphate (calcium, iron, and aluminum), in addition to synthesizing hydrolytic enzymes that, in addition to their protective effect against phytopathogens, also contribute to plant development. These effects were evidenced in the growth-promotion assays, particularly through the significant stimulation of *C. chinense* root system development.

The mechanisms of phosphate solubilization and siderophore production play a fundamental role in promoting plant growth by increasing the availability of essential nutrients in the rhizosphere. The solubilization of inorganic phosphates converts insoluble forms of phosphorus into forms that can be readily absorbed by plants, while siderophores sequester iron under conditions of low availability, whereas siderophore production increases iron uptake by plants and limits iron availability to phytopathogens. These mechanisms highlight the multifunctional role of *Streptomyces*, with promising applications in both biocontrol and biofertilization, as demonstrated in recent studies [56,57,58].

Taken together, the results obtained in this study reinforce the effectiveness of *Streptomyces* in controlling phytopathogenic fungi and promoting growth in *C. chinense* crops, aligning with several recent studies that demonstrate their ability as promising biocontrol agents for sustainable agriculture. The characterization of new strains with both antifungal and growth-promoting activity, particularly those isolated from underexplored environments such as the Amazon, represents a significant contribution to the development of biological alternatives to conventional agrochemicals.

## 5. Conclusions

This study provides novel insights by thoroughly characterizing the multifunctional potential of two *Streptomyces* strains, including a putative new species (APUR 32.5) and *S. murinus* (MPUR 40.3), originally isolated from the highly biodiverse Amazonian sediments. Our findings strongly support the hypothesis that these underexplored ecosystems are rich sources of microorganisms that possess unique and potent biotechnological attributes, which may be applicable in sustainable agricultural solutions. These Amazonian isolates demonstrated robust, broad-spectrum inhibitory capacity against all the tested *Colletotrichum* species in vitro. More critically, they showed significant efficacy in reducing the incidence of postharvest anthracnose in *Capsicum chinense* fruits, with *S. murinus* MPUR 40.3 achieving a remarkable 95% reduction and *Streptomyces* sp. APUR 32.5 achieving a notable 39.25% reduction. Mechanistic insights from scanning electron microscopy revealed distinct antifungal actions, with MPUR 40.3 primarily inducing conidial lysis during early infection and APUR 32.5 causing hyphal degradation. In addition to biocontrol, these isolates exhibited plant growth-promoting traits, including the production of hydrolytic enzymes and siderophores, as well as phosphate solubilization. Their demonstrated ecophysiological versatility (tolerance to broad ranges of temperature and pH) further enhances their applicability. Collectively, these multifunctional Amazonian *Streptomyces* isolates represent promising, sustainable candidates for integrated phytopathogen management and enhanced crop productivity in agriculture. Future studies employing metabolomic approaches will aim to elucidate the molecular mechanisms associated with the antifungal activity of these strains. In addition, their efficacy under field conditions and their potential for the development of commercial formulations as bioinoculants and biocontrol agents will be evaluated.

## Data Availability

The original contributions presented in this study are included in the article/Supplementary Materials. Further inquiries can be directed to the corresponding author.

## Supplementary Materials

The following supporting information can be downloaded at: https://www.mdpi.com/article/10.3390/microorganisms13122713/s1, Table S1. Composition of the culture media used in this study for the morphological characterization of *Streptomyces* strains and antagonism analyses. Table S2. Sequences of the primers used for partial amplification of the genes *atpD*, *gyrB*, *recA*, *rpoB*, and *trpB*, with their respective annealing temperatures and expected PCR product sizes. Table S3. Sequence data and GenBank accession numbers for *Streptomyces* strains used in this study. Table S4. Phytopathogens of the genus *Colletotrichum* used in antagonism assays with *Streptomyces* sp. APUR 32.5 and *Streptomyces murinus* MPUR 40.3. Table S5. Composition of the selective culture media used to evaluate the solubilization capacity of different phosphate sources and the production of siderophores by *Streptomyces* isolates. Table S6. Composition of the culture media used in the enzymatic activity assays and the methods for detecting hydrolysis halos. Table S7. Percentage of digital DNA-DNA hybridization (dDDH) values calculated using formula d4 among genomes of strains MPUR 40.3 and APUR 32.5 compared with phylogenetically closest *Streptomyces* species using the Type Strain Genome Server (TYGS).
