# Supplementary material for: Streptomyces Strains from Amazonian Sediments as Plant Growth Promoters and Biocontrol Agents of Anthracnose in Postharvest Capsicum chinense
Source: Microorganisms. 2025 Nov 28;13(12):2713. doi: 10.3390/microorganisms13122713 (PMC12734931; doi:10.3390/microorganisms13122713)
Supplement: Supplementary file 1 [file microorganisms-13-02713-s001.zip › microorganisms-3948456-supplementary.pdf]

Article

Streptomyces Strains from Amazonian Sediments as Plant Growth Promoters and Biocontrol Agents of Anthracnose in Postharvest *Capsicum chinense*

Ingride Jarline Santos da Silva [1,2], Thiago Fernandes Sousa [1,2], Thayná Marães de Souza [2], Beatriz Miranda Gomes [1], Rudi Emerson de Lima Procópio [3], Aleksander Westphal Muniz [2], Rogério Eiji Hanada [4], Hector Henrique Ferreira Koolen [3] and Gilvan Ferreira da Silva [2,*]

Table S1. Composition of the culture media used in this study for the morphological characterization of *Streptomyces* strains and antagonism analyses.

Culture Media	Composition (per 1 L of distilled water)
**ISP1**	5 g tryptone; 3 g yeast extract; 20 g agar
**ISP2**	4 g yeast extract; 10 g malt extract; 4 g dextrose; 20 g agar
**ISP3**	20 g oatmeal; 18 g agar
**LB**	10 g peptone; 5 g yeast extract; 5 g NaCl; 20 g agar
**ACA**	10 g starch; 0.3 g casein; 2 g KNO_3; 2 g NaCl; 2 g K_2HPO_4; 0.05 g $MgSO_4$; 1.02 g $CaCO_3$; 0.01 g $FeSO_4$; 15 g agar
**SYEP**	0.25 g yeast extract; 0.5 g K_2HPO_4; 18 g agar
**V8**	200 mL V8 juice; 4.5 g $CaCO_3$; 18 g agar; 800 mL distilled water
**BDA**	200 g potato; 20 g dextrose; 15 g agar
**SNA**	1 g KH_2PO_4; 1 g KNO_3; 0.5 g $MgSO_4$; 0.5 g KCl; 0.2 g glucose; 0.2 g sucrose; 20 g agar
**AA**	17 g agar
**AIA**	2 g sodium caseinate; 0.1 g L-asparagine; 4 g sodium propionate; 0.5 g K_2HPO_4; 0.1 g $MgSO_4$; 0.001 g $FeSO_4$; 15 g agar; 5 mL glycerol
**Rice Agar**	40 g rice; 0.04 g yeast extract; 0.02 g K_2HPO_4

Table S2. Sequences of the primers used for partial amplification of the genes *atpD*, *gyrB*, *recA*, *rpoB*, and *trpB*, with their respective annealing temperatures and expected PCR product sizes.

Gene	Primer	Sequences	Annealing temperature (°C)	Pb
atpD	atpDF<br>atpDR	ACCAAGGGCAAGGTGTTCAA<br>GCCGGGTAGATGCCCTTCTC	63	998
gyrB	gyrBF-1<br>gyrBR-4	GAGGTCGTGCTGACCGTGCTGCA<br>CGCTCCTTGTCCTCGGCCTC	65	1305
recA	recAF<br>recAR	ACAGATTGAACGGCAATTCG<br>ACCTTGTTCTTGACCACCTT	60	913
rpoB	rpoBF1<br>rpoBR1	TTCATGGACCAGAACAACC<br>CGTAGTTGTGACCCTCCC	65	994
trpB	trpBF<br>trpBR	GGCTCACACAAGATCAACAA<br>TCGATGGCCGGGATGATGCC	65	822

Table S3. Sequence data and GenBank accession numbers for *Streptomyces* strains used in this study.

Species	Strain	atpD	gyrB	trpB	rpoB	recA
Streptomyces sp.	**APUR 32.5**	**PX502295**	**PX502296**	**PX502297**	**PX502299**	**PX502298**
Streptomyces murinus	**MPUR 40.3**	**PX502290**	**PX502291**	**PX502292**	**PX502293**	**PX502294**
Streptomyces murinus	NRRL B-2286	KT384651.1	KT385001.1	KT389320.1	KT388971.1	KT385352.1
Streptomyces glaucescens	NRRL B-2706	KT384565.1	HQ823590.1	KT389234.1	KT388884.1	KT385263.1
Streptomyces pharetrae	NRRL B-24333	KT384683.1	KT385033.1	KT389353.1	KT389004.1	KT385384.1
Streptomyces spinoverrucosus	NRRL B-16932	KT384725.1	KT385074.1	KT389394.1		KT385426.1
Streptomyces galbus	NRRL B-2283	KT384560.1	KT384909.1	KT389229.1	KT388879.1	KT385258.1
Streptomyces inusitatus	NRRL B-16929	KT384603.1	KT384952.1	KT389272.1	KT388923.1	KT385302.1
Streptomyces longwoodensis	NRRL B-16923	KT384629.1	KT384978.1	KT389298.1	KT388949.1	KT385329.1
Streptomyces thermospinosisporus	NRRL B-24318	KT384737.1	KT385086.1	KT389406.1	KT389057.1	KT385439.1
Streptomyces echinoruber	NRRL 8144	KT384540.1	KT384889.1	KT384540.1	KT388859.1	KT385237.1
Streptomyces thermoviolaceus	NRRL B-12374	KT384738.1	KT385087.1	KT389407.1	KT389058.1	KT385440.1
Streptomyces albogriseolus	NRRL B-1305	KT384453.1	KT384802.1	KT389122.1	KT388772.1	KT385150.1
Streptomyces wellingtoniae	NRRL B-1503	KT384761.1	KT385109.1	KT389430.1	KT389081.1	KT385463.1
Streptomyces phaeoluteichromatogenes	NRRL B-5799	KT384680.1	KT385030.1	HG423654.1	HG423678.1	KT385381.1
Streptomyces misionensis	NRRL B-3230	KT384647.1	KT384996.1	KT389316.1	KT388967.1	KT385347.1
Streptomyces griseofuscus	NRRL B-5429	KT384579.1	KT384928.1	KT389248.1	KT388898.1	KT385277.1
Streptomyces costaricanus	NRRL B-16897	KT384774.1	KT385122.1	KT389443.1	KT389094.1	KT385476.1
Streptomyces viridiviolaceus	NRRL B-12182	KT384755.1	KT385103.1	KT389424.1	KT389075.1	KT385457.1
Streptomyces grisestramineus	NRRL B-5422	KT384585.1	KT384934.1	KT389254.1	KT388905.1	KT385283.1
Streptomyces graminearus	NRRL B-16369	KT384573.1	KT384922.1	KT389242.1	KT388892.1	KT385271.1
Streptomyces chromofuscus	NRRL B-12175	KT384509.1	KT384858.1	KT389178.1	KT388828.1	KT385206.1

Streptomyces levis	NRRL B-16370	KT384621.1	KT384970.1	HG423660.1	HG423682.1	KT385320.1
Streptomyces indiaensis	NRRL B-24311	KT384601.1	KT384950.1	KT389270.1	KT388921.1	KT385300.1
Streptomyces massasporeus	NRRL B-3300	KT384636.1	KT384985.1	KT389305.1	KT388956.1	KT385336.1
Streptomyces asoensis	NRRL B-16592	KT384493.1	KT384842.1	KT389162.1	KT388812.1	KT385190.1
Streptomyces lateritius	NRRL B-5349	KT384616.1	KT384965.1	KT389285.1	KT388936.1	KT385315.1
Streptomyces zaomyceticus	NRRL B-2038	KT384771.1	KT385119.1	KT389440.1	KT389091.1	KT385473.1
Streptomyces bikiniensis	NRRL B-2690	KT384486.1	KT384835.1	KT389155.1	KT388805.1	KT385183.1
Streptomyces gardneri	NRRL B-5615	KT384562.1	KT384911.1	KT389231.1	KT388881.1	KT385260.1
Streptomyces venezuelae	NRRL ISP-5230	KT384746.1	KT385094.1	KT389415.1	KT389066.1	KT385448.1
Streptomyces litmocidini	NRRL B-3635	KT384625.1	KT384974.1	KT389294.1	KT388945.1	KT385325.1
Streptomyces showdoensis	NRRL B-12430	KT384722.1	KT385071.1	KT389391.1	KT389043.1	KT385423.1
Streptomyces violaceorectus	NRRL B-12181	KT384750.1	KT385098.1	KT389419.1	KT389070.1	KT385452.1
Streptomyces viridobrunneus	NRRL B-24332	KT384753.1	KT385101.1	KT389422.1	KT389073.1	KT385455.1
Streptomyces roseoviridis	NRRL B-2730	KT384711.1	KT385061.1	KT389380.1	KT389032.1	KT385412.1
Streptomyces filamentosus	NRRL B-2114	KT384547.1	KT384896.1	KT389216.1	KT388866.1	KT385245.1
Streptomyces omiyaensis	NRRL B-1587	KT384671.1	KT385021.1	KT389340.1	KT388991.1	KT385372.1
Streptomyces roseolus	NRRL B-5424	KT384707.1	KT385057.1	KT389376.1	KT389028.1	KT385408.1
Streptomyces roseofulvus	NRRL B-2729	KT384706.1	KT385056.1	KT389375.1	KT389027.1	KT385407.1
Streptomyces tanashiensis	NRRL B-2606	KT384656.1	KT385006.1	KT389325.1	KT388976.1	KT385357.1
Streptomyces netropsi	NRRL B-1831	KT384609.1	KT384958.1	KT389278.1	KT388929.1	KT385308.1

Table S4. Phytopathogens of the genus *Colletotrichum* used in antagonism assays with *Streptomyces* sp. APUR 32.5 and *Streptomyces murinus* MPUR 40.3.

N°	Phytopathogen	Strain	Host
1	*Colletotrichum siamense*	Coll 2N	*Synedrella nodiflora*
2	*Colletotrichum theobromicola*	1809	*Allium schoenoprasum*
3	*Colletotrichum brevisporum*	2787	*Capsicum chinense* (fruit)
4	*Colletotrichum spaethianum*	2908	*Allium schoenoprasum*
5	*Colletotrichum scovillei*	2910	*Capsicum chinense* (fruit)
6	*Colletotrichum guaranicola*	2939	*Paullinia cupana* var. *sorbilis* (leaf)
7	*Colletotrichum* sp.	2973	Carica papaya (fruit)

Table S5 – Composition of the selective culture media used to evaluate the solubilization capacity of different phosphate sources and the production of siderophores by *Streptomyces* isolates.

Activity evaluated	Culture media	Composition (g/L)	Indicator of solubilization/production	References
**Calcium phosphate solubilization**	NBRIP medium	Glicose (10,0); $Ca_3(PO_4)_2$ (5,0); $MgCl_2{\cdot}6H_2O$ (5,0); $MgSO_4{\cdot}7H_2O$ (0,25); KCl (0,2); $(NH_4)_2SO_4$ (0,1); Ágar (15,0); pH 7	Formation of a clear halo around the colony	Nautiyal (1999)
**Aluminum phosphate solubilization**	Modified NBRIP medium	Glucose (10.0 g); $FePO_4$ (5.0 g); $MgCl_2{\cdot}6H_2O$ (5.0 g); $MgSO_4{\cdot}7H_2O$ (0.25 g); KCl (0.2 g); $(NH_4)_2SO_4$ (0.1 g); Agar (15.0 g); pH 7	Formation of a clear halo around the colony	Adaptado de Nautiyal (1999)
**Iron phosphate solubilization**	Modified NBRIP medium	Glucose (10,0); $FePO_4$ (5,0); $MgCl_2{\cdot}6H_2O$ (5,0); $MgSO_4{\cdot}7H_2O$ (0,25); KCl (0,2); $(NH_4)_2SO_4$ (0,1); Ágar (15,0); pH 7	Formation of a clear halo around the colony	Adaptado de Nautiyal (1999)
**Siderophore production**	Meio CAS-ágar	*CAS solution (100 mL); PIPES (30.24); Trace element solution** (10 mL); Casamino acids (0.5 g); Asparagine (0.5 g); Agar (15.0 g); pH 6.8**	Formation of an orange halo around the colony	Schwyn & Neilands (1987)

Table S6. Composition of the culture media used in the enzymatic activity assays and the methods for detecting hydrolysis halos.

Activity evaluated	Culture media	Detection	References
**Amylase**	5 g peptone; 5 g NaCl; 5 g meat extract; 10 g dextrose; 2 g starch; 20 g agar; 1 L distilled water	10 mL of 0.3% (v/v) Lugol's solution for 5 min. Formation of halos around the isolate.	Hankin & Anagnostakis (1975)
**Cellulase**	1 g KH_2PO_4; 0.5 g $MgSO_4 \cdot 7H_2O$; 0.5 g NaCl; 0.01 g $FeSO_4 \cdot 7H_2O$; 0.01 g $MnSO_4 \cdot H_2O$; 0.3 g NH_4NO_3; 10 g carboxymethylcellulose; 12 g agar; 1 L distilled water	10 mL of Congo Red (0.1% v/v) for 15 min, followed by 10 mL of 1 M NaCl for an additional 15 min. Formation of halos around the bacterium.	Ariffin et al. (2006)
**Chitinase**	10 g colloidal chitin; 0.2 g KCl; 0.2 g $MgSO_4 \cdot 7H_2O$; 1 g $NH_4H_2PO_4$; 1 L distilled water	Formation of halos around the colony.	Roberts & Selitrennikoff (1988)
**Lipase**	10 g peptone; 5 g NaCl; 0.1 g $CaCl_2 \cdot 2H_2O$; 20 g agar; 10 mL Tween 20; 1 L distilled water	Formation of whitish halos around the colony.	Sierra (1957)
**Protease**	2 g skim milk powder; 5 g peptone; 3 g yeast extract; 15 g agar; 1 L distilled water	Formation of halos around the colony.	Hankin & Anagnostakis (1975)

Table S7. Percentage of digital DNA-DNA hybridization (dDDH) values calculated using formula d4 among genomes of strains MPUR 40.3 and APUR 32.5 compared with phylogenetically closest *Streptomyces* species using the Type Strain Genome Server (TYGS).

Query strain	Subject strain	d4	C.I. (d4, in %)	G+C content difference (in %)
**MPUR 40.3**	*Streptomyces murinus* DSM 41827	84.5	[81.8 - 86.9]	0.04
**MPUR 40.3**	*Streptomyces graminearus* JCM 6923	75.2	[72.2 - 78.0]	0.04
**MPUR 40.3**	*Streptomyces murinus* NRRL B-5429	71.6	[68.5 - 74.4]	0.07
**MPUR 40.3**	*Streptomyces murinus* DSM 40710	71.5	[68.5 - 74.4]	0.08
**MPUR 40.3**	*Streptomyces murinus* DSM 40191	71.5	[68.4 - 74.3]	0.14
**APUR 32.5**	*Streptomyces murinus* DSM 41827	55.1	[52.3 - 57.8]	0.53
**APUR 32.5**	*Streptomyces* sp. MPUR40.3	55.0	[52.3 - 57.7]	0.49
**APUR 32.5**	*Streptomyces graminearus* JCM 6923	54.1	[51.4 - 56.8]	0.46
**APUR 32.5**	*Streptomyces murinus* NRRL B-5429	53.8	[51.1 - 56.5]	0.57
**APUR 32.5**	*Streptomyces murinus* DSM 40710	53.7	[51.0 - 56.4]	0.57
**APUR 32.5**	*Streptomyces murinus* DSM 40191	53.6	[50.9 - 56.3]	0.64
